# Plasma modes in capacitively coupled superconducting nanowires

**DOI:** 10.3762/bjnano.13.24

**Published:** 2022-03-04

**Authors:** Alex Latyshev, Andrew G Semenov, Andrei D Zaikin

**Affiliations:** 1 I.E. Tamm Department of Theoretical Physics, P.N. Lebedev Physical Institute, 119991 Moscow, Russia; 2 National Research University Higher School of Economics, 101000 Moscow, Russia; 3 Department of Physics, Moscow Pedagogical State University, 119435 Moscow, Russia; 4 Institute for Quantum Materials and Technologies, Karlsruhe Institute of Technology (KIT), 76021 Karlsruhe, Germany

**Keywords:** plasma modes, quantum fluctuations, quantum phase slips, superconducting nanowires

## Abstract

We investigate plasma oscillations in long electromagnetically coupled superconducting nanowires. We demonstrate that in the presence of inter-wire coupling plasma modes in each of the wires get split into two “new” modes propagating with different velocities across the system. These plasma modes form an effective dissipative quantum environment interacting with electrons inside both wires and causing a number of significant implications for the low-temperature behavior of the systems under consideration.

## Introduction

Physical properties of ultrathin superconducting nanowires differ strongly from those of bulk superconductors owing to a prominent role of fluctuation effects in a reduced dimension [1–3]. Such fluctuations cause a reduction of the superconducting critical temperature [4] and yield a negative correction to the mean field value of the order parameter Δ_0_. In particular, at *T*→0 for the absolute value of the order parameter |Δ| in superconducting nanowires one finds [5]:


[1]

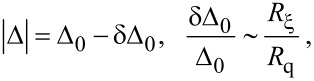



where *R*_ξ_ is the normal-state resistance of the wire segment of length equal to the superconducting coherence length ξ and *R*_q_ = 2π/*e*^2^ ≃ 25.8 kΩ is the quantum resistance unit. For generic metallic nanowires one typically has *R*_ξ_ ≪ *R*_q_, implying that fluctuation correction to the mean value of the superconducting order parameter (Equation 1) remains weak and in the majority of cases can be neglected.

Is the condition *R*_ξ_/*R*_q_ ≪ 1 sufficient to disregard fluctuation effects in superconducting nanowires? The answer to this question is obviously negative since even in this limit fluctuations of the phase φ(*x*,*t*) of the order parameter Δ = |Δ|exp(*i*φ) survive being essentially decoupled from those of the absolute value |Δ|. Such phase fluctuations are intimately related to sound-like plasma modes [6–7] (the so-called Mooij–Schön modes), which can propagate along the wire playing the role of an effective quantum dissipative environment for electrons inside the wire. The frequency spectrum of this effective environment is similar to that of the celebrated Caldeira–Leggett model [8], which is widely employed in order to account for both quantum dissipation and quantum decoherence in normal [9–10] and superconducting [11–12] metallic structures, see also the book [1] for an extensive review on this issue.

The presence of Mooij–Schön plasma modes is an important feature inherent to long superconducting nanowires that leads to a number of interesting effects. One of them is the theoretically predicted [13–14] and experimentally observed [15–16] smearing of the square-root singularity in the density of states (DOS) near the superconducting gap accompanied by a non-vanishing tail in DOS at subgap energies. Mooij–Schön plasmons also mediate the interaction between quantum phase slips (QPS) [1–2,17–18] causing Berezinskii–Kosterlitz–Thouless-like [17] and Schmid-like [19–21] quantum phase transitions in structures involving superconducting nanowires.

In this work we are going to investigate propagation of plasma modes in a system of two long capacitively coupled superconducting nanowires. We are going to demonstrate that in the presence of electromagnetic interaction between the wires their plasma modes get split into a pair of “new” modes propagating along the system with two different velocities. This effect may have various implications for the low-temperature behavior of the structures under consideration.

## Results and Discussion

**Figure 1:**
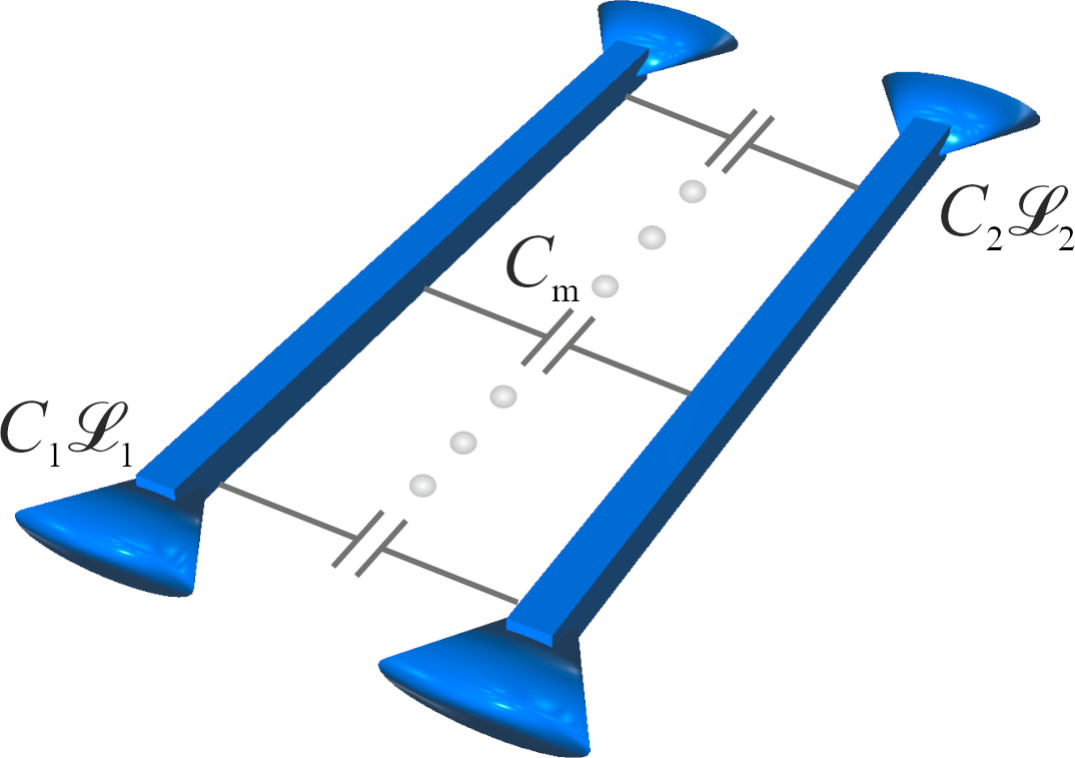
The system of two capacitively coupled superconducting nanowires.

Consider a system composed of two long parallel to each other superconducting nanowires. This structure is schematically depicted in Figure 1. The wires are characterized by kinetic inductances 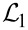 and 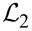 (times unit wire length) and geometric capacitances *C*_1_ and *C*_2_ (per unit length). In the absence of any interaction between the wires they represent two independent transmission lines where low-energy plasma excitations propagate with velocities 
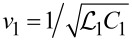
 and 
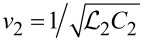
, respectively, in the first and the second wires.

Note that the wires can be treated as independent only provided that they are located far from each other. If, on the contrary, the distance between the wires becomes sufficiently short they develop electromagnetic coupling even though there exists no direct electric contact between them. In this case each fluctuation associated with an electromagnetic pulse in the first wire induces an electromagnetic perturbation in the second one and vice versa. Accordingly, propagation of plasma modes along the wires gets modified and is not anymore described by two independent velocities *v*_1_ and *v*_2_. The task at hand is to investigate the effect of electromagnetic coupling on plasma excitations in the system of two superconducting nanowires.

To this end, we will routinely model electromagnetic coupling between the wires by introducing mutual geometric inductance 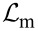 and capacitance *C*_m_ for these wires. All geometric inductances for ultrathin superconducting wires are typically much smaller than kinetic ones and, hence, 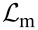 can be safely neglected as compared to 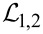. On the contrary, the mutual capacitance *C*_m_ can easily reach values comparable with *C*_1_*_,_*_2_ and for this reason it needs to be explicitly accounted for within the framework of our consideration.

As a result, making use of the microscopic effective action analysis [17–18,22] we arrive at the following Hamiltonian that includes both electric and magnetic energies of our superconducting nanowires [23–24]


[2]

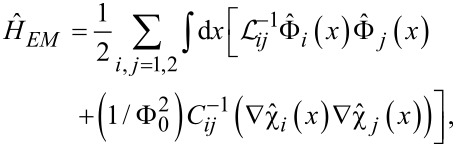



where *x* denotes the coordinate along the nanowires,


[3]

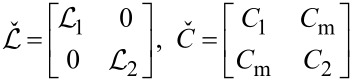



(*C*_m_ *< C*_1_*_,_*_2_) are the inductance and capacitance matrices and Φ_0_ = π/*e* is the superconducting flux quantum (here and below we set Planck constant ℏ, speed of light *c* and Boltzmann constant *k*_B_ equal to unity).

The Hamiltonian in Equation 2 is expressed in terms of the dual operators 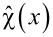 and 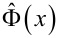 [25] obeying the canonical commutation relation


[4]

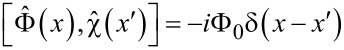



and are linked to the charge density and the phase operators 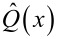 and 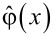 as


[5]

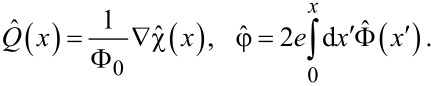



Physically, 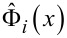 represents the magnetic flux operator, while the operator 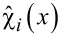 is proportional to that for the total charge 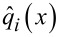 that has passed through the point *x* of the *i*-th wire up to the same time moment *t*, that is, 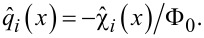


As we already pointed out above, in the case of two capacitively coupled wires any perturbation that occurs in one of the wires generates charge redistribution and voltage pulses in both wires. The corresponding voltage drop in these wires 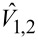 can be expressed in terms of the local charge operators by means of the following equation [23]:


[6]

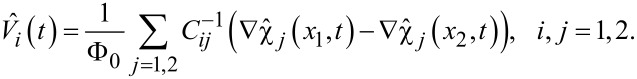



In what follows, it will be convenient for us to go over to the phase representation and to express the equation of motion for the phase perturbations φ_1_*_,_*_2_ in both wires in the form of


[7]

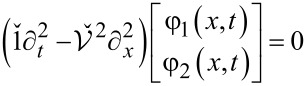



that follows directly from the Hamiltonian for our structure (Equation 2). Here 
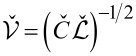
is the velocity matrix that accounts for plasma modes propagating along the wires.

In order to evaluate the velocities of plasma modes in the presence of electromagnetic coupling between the wires it is necessary to diagonalize the velocity matrix 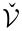 and to determine its eigenvalues *v*_±_. Making use of Equation 3 after a trivial algebra we obtain


[8]

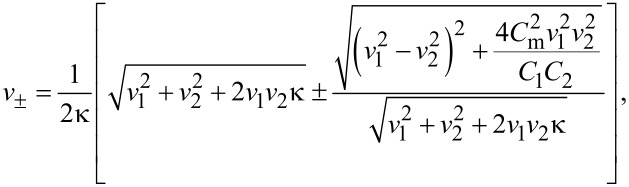



where we defined 
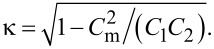


Equation 8 represents the central result of our present work. It demonstrates that in the presence of electromagnetic coupling plasma modes in each of the wires are split into two “new” modes being common for both wires and propagating along them with velocities *v*_±_. As we expected, no independent plasma modes in each of the wires could exist in this case. Only in the absence of inter-wire interaction (i.e., for κ = 1) Equation 8 yields *v*_+_ = *v*_1_ and *v*_−_ = *v*_2_.

In the case of identical wires with *C*_1_ = *C*_2_ = *C*, 
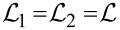
 and *v*_1_ = *v*_2_ = *v* the result (Equation 8) reduces to a particularly simple form


[9]

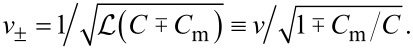



Provided the parameters of both wires differ in such a way that one of the unperturbed velocities strongly exceeds the other one, *v*_1_ ≫ *v*_2_, Equation 8 yields


[10]

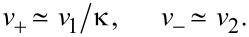



Equation 8–Equation 10 demonstrate that one of the plasma modes may propagate much faster than any of such modes in the absence of inter-wire interaction. This situation can be realized provided the wires are located close enough to each other in which case the cross-capacitance *C*_m_ may become of the same order as *C*_1_*_,_*_2_ implying κ ≪ 1.

Provided the wires are thick enough, the low-energy Hamiltonian in Equation 2 is sufficient. However, for thinner wires one should also take into account the effect of quantum phase slips [1–2,17–18], which correspond to a fluctuation-induced local temporal suppression of the superconducting order parameter inside the wire accompanied by the phase slippage process and quantum fluctuations of the voltage in the form of pulses. Here, it will be sufficient for our purposes to account for QPS effects only in the first wire and ignore these effects in the second one. In this case, the Hamiltonian in Equation 2 should be replaced by that for an effective sine-Gordon model [25]


[11]

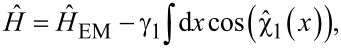



where the last term describes QPS effects in the first wire and γ_1_ defines the QPS amplitude (per unit wire length) in this wire. In simple terms, the last term in Equation 11 can be treated as a linear combination of creation (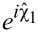) and annihilation (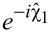) operators for the flux quantum Φ_0_ and accounts for tunneling of such flux quanta Φ_0_ across the first wire.

**Figure 2:**
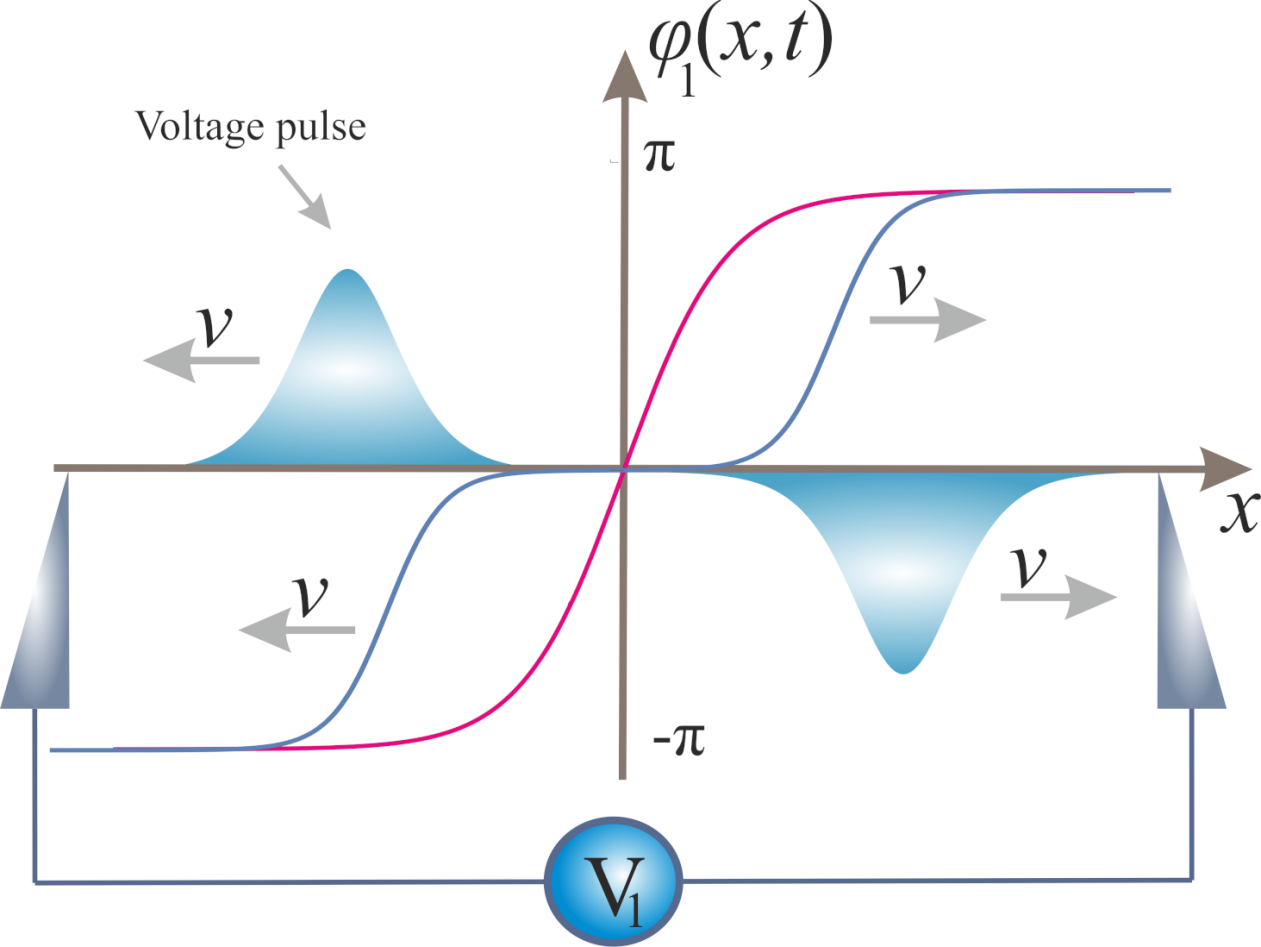
Time-dependent phase configurations describing a QPS event at *t* = 0 (red) and *t >* 0 (blue) together with propagating voltage pulses generated by this QPS event in a single superconducting nanowire.

It is well known that any QPS event causes redistribution of charges inside the wire and generates a pair of voltage pulses propagating simultaneously in opposite directions along the wire. Assume that a QPS event occurs at the initial time moment *t* = 0 at the point *x* = 0 inside the first wire. This event corresponds to the phase jump by 2π, as it is shown in Figure 2. Provided the first wire is electromagnetically decoupled from the second one, at *t >* 0, voltage pulses originating from this QPS event will propagate with the velocity 
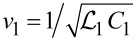
 along the first wire, see Figure 2. Obviously, the second wire remains unaffected.

**Figure 3:**
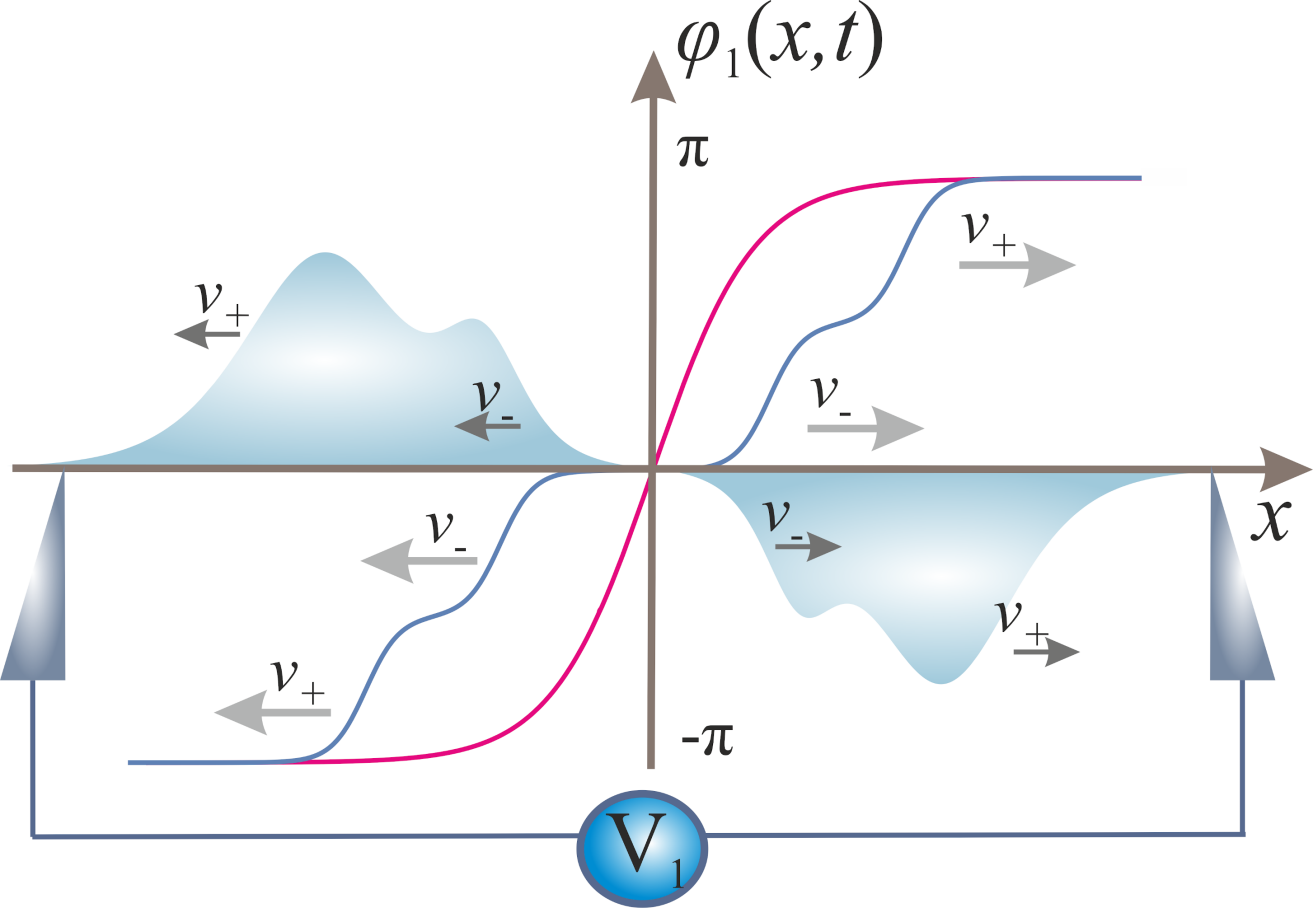
The same as in Figure 1 in the first of the two capacitively coupled superconducting nanowires. Each of the voltage pulses is split into two propagating with different velocities *v*_±_.

**Figure 4:**
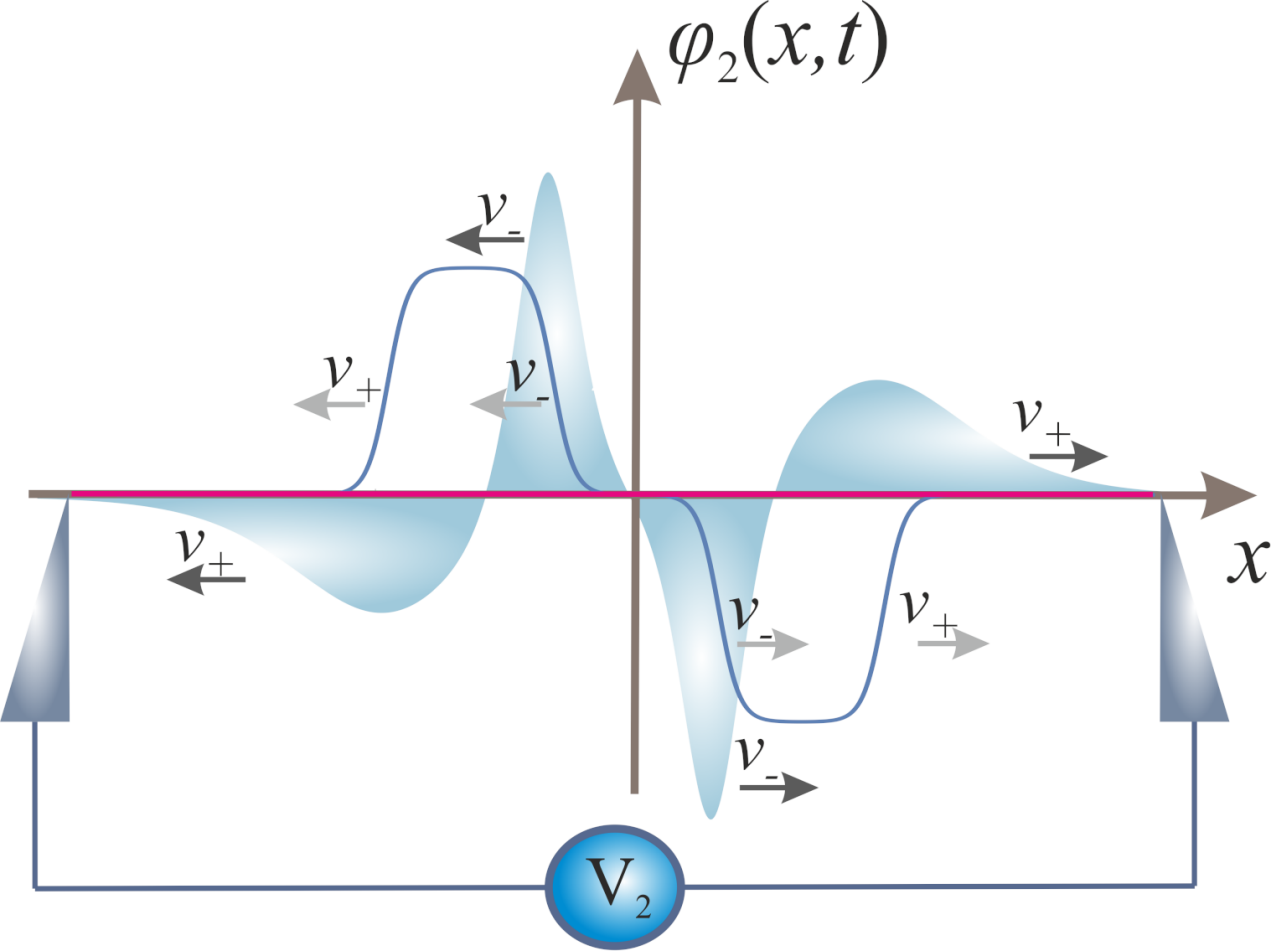
Time-dependent phase configurations at *t* = 0 (red) and *t >* 0 (blue) together with propagating voltage pulses in the second of the two capacitively coupled superconducting nanowires generated by a QPS event in the first one.

Let us now “turn on” capacitive coupling between the wires. In this case, quantum phase slips in one of the wires generate voltage pulses already in both wires. Resolving Equation 7 together with proper initial conditions corresponding to a QPS event, we arrive at the following picture, summarized in Figure 3 and Figure 4. In the first wire each of the two voltage pulses propagating in opposite directions is now, in turn, split into two pulses of the same sign moving with different velocities *v*_+_ and *v*_−_, as it is illustrated in Figure 3. Voltage pulses generated in the second wire by a QPS event in the first one have a different form. There are also two pairs of pulses propagating in opposite directions with velocities *v*_+_ and *v*_−_ along the second wire. However, the signs of voltage pulses moving in the same direction are now opposite to each other, cf. Figure 4. This result clearly illustrates specific features of voltage fluctuations induced in the second wire by a QPS event in the first wire: Such fluctuations are characterized by zero average voltage and non-vanishing voltage noise [24].

## Conclusion

In this work we have investigated plasma oscillations in capacitively coupled superconducting nanowires. We have shown that in such structures there exist two plasma modes propagating with different velocities along the wires. We have explicitly evaluated these velocities and demonstrated that these plasma modes are the same for both wires forming a single effective dissipative quantum environment interacting with electrons inside the structure. Our results might have significant implications for the low-temperature behavior of coupled superconducting nanowires. For instance, electron DOS in each of the wires can be affected by fluctuations in a somewhat different manner as compared to the noninteracting case [13–16]. Likewise, the logarithmic interaction between different quantum phase slips mediated by such plasma modes gets modified, implying a shift of the superconductor–insulator quantum phase transition in a way to increase the tendency towards localization of Cooper pairs [23]. Further interesting effects are expected that can be related to the correlated behavior of quantum phase slips in different superconducting nanowires. This problem, however, goes beyond the scope of the present paper and will be studied elsewhere.
